# Tracking of a Dietary Pattern and Its Components over 10-Years in the Severely Obese

**DOI:** 10.1371/journal.pone.0097457

**Published:** 2014-05-19

**Authors:** David J. Johns, Anna Karin Lindroos, Susan A. Jebb, Lars Sjöström, Lena M. S. Carlsson, Gina L. Ambrosini

**Affiliations:** 1 Medical Research Council Human Nutrition Research, Elsie Widdowson Laboratory, Cambridge, United Kingdom; 2 Institute of Medicine, University of Gothenburg, Gothenburg, Sweden; 3 The National Food Administration, Uppsala, Sweden; University of East Anglia, United Kingdom

## Abstract

Understanding how dietary intake changes over time is important for studies of diet and disease and may inform interventions to improve dietary intakes. We investigated how a dietary pattern (DP) tracked over 10-years in the Swedish Obese Subjects (SOS) study control group. Dietary intake was assessed at multiple time-points in 2037 severely obese individuals (BMI 41±4 kg/m^2^). Reduced rank regression was used to derive a dietary pattern using dietary energy density (kJ/g), saturated fat (%) and fibre density (mg/kJ) as response variables and score respondents at each follow-up. Tracking coefficients for the DP, its key foods and macronutrient response variables and corrected for time-dependent and time-independent covariates were calculated using generalised estimating equations to take into account all available data. The DP tracking coefficient was moderate for women (0.40; 95% CI: 0.38–0.42) and men (0.38; 95% CI: 0.35–0.41). Of the eleven foods key to this DP, fruit and vegetable intakes had the strongest tracking coefficient for both sexes. Fast food and candy had the lowest tracking coefficients for women and men respectively. Scores for an energy dense, high saturated fat, low fibre density DP appear moderately stable over a 10-year period in this severely obese population. Furthermore, some food groups appear more amenable to change while others, often the most healthful, appear more stable and may require intervention before adulthood.

## Introduction

In epidemiological literature tracking is used to describe the stability of the longitudinal development of a certain outcome variable [1]. Understanding how dietary intake changes over time i.e. its stability or tracking, can help to in identify targets for interventions to improve diet quality. Individual food groups with strong tracking of dietary intake are likely less amenable to change than those with weak tracking. Tracking of dietary intake over time in different populations also has methodological implications for cohort design and dietary assessment.

Little is known about longitudinal tracking of dietary intake in adults. Some work has previously been conducted looking at the tracking of food intake in children and adolescents [2], [3], [4], [5], [6] and their transition into adulthood [7], [8]. Few studies have considered changes in dietary patterns over time [9], [10], [11].

To our knowledge, no studies examining changes in dietary intake over time have been conducted in obese populations. This is despite weight-loss attempts being more prevalent and frequent in this group [12], [13]. Understanding the role of tracking in a severely obese population will provide an insight into the opportunities for lifestyle interventions in this population.

Dietary patterns allow us to account for total dietary intake and the various combinations of food and nutrients typically consumed [14]. However, few studies have considered how dietary patterns change over time in adults using more than two time points [15], [16], [17] and even fewer have made use of all the available data in longitudinal analysis [16].

Reduced rank regression (RRR) is a dietary pattern method that balances hypothesis and exploratory driven aspects [18], [19]. It creates patterns in food groups that optimise the amount of variation in a set of variables chosen by the researcher using a priori knowledge.

The aim of this study was to investigate tracking of dietary intake in a severely obese population over a 10-year period, in particular, a hypothesis driven dietary pattern identified using RRR. The tracking of intakes of selected food groups and nutrients was also examined.

## Materials and Methods

Ethics statement: Written Informed consent was obtained for all study participants. The Swedish Obese Subjects (SOS) study has been conducted according to the principles expressed in the Declaration of Helsinki. The SOS study protocol was approved by the Research Ethics Committee of University of Gothenburg and seven other Swedish regional ethics review boards, each harbouring one or several of the involved study sites. The SOS trial has been registered in the ClinicalTrials.gov registry (NCT01479452, http://clinicaltrials.gov/ct2/show/NCT01479452?term).

Data were from the Swedish Obese Subjects Study (SOS) study. Full details of the study have been reported elsewhere [20]. In brief, SOS is a prospective intervention study investigating the impact of bariatric surgery on morbidity and mortality in severely obese adults. SOS recruited 6905 severely obese individuals between 1987 and 2001 in Sweden, all of whom underwent health examinations and completed multiple questionnaires including diet. Of this registry population, 2010 individuals chose to have bariatric surgery and were matched with 2037 control who received no standardised treatment [20].

The current analysis uses data from the control group only (n = 2037). Participants completed medical, dietary and health related quality-of-life questionnaires as well as health examinations at registration, baseline (t = 0), 0.5, 1, 2, 3, 4, 6, 8, and 10-years. Data collected up to 16^th^ June 2009 is included.

### Dietary Assessment

Dietary data were collected using a validated, semi-quantitative diet questionnaire. This questionnaire was adapted from a diet history interview developed for the general population in Sweden, based on clinical experience of the problematic eating characteristics of obese individuals. The questionnaire included 50 questions with additional sub-questions intended to add detail and clarification. The questionnaire, described in more detail elsewhere [21], has been validated in both obese and non-obese groups [21], [22]. The correlations between the questionnaire and food records for reported intakes of dietary fibre, saturated fat and total energy were 0.59, 0.62 and 0.45 respectively [21]. The reproducibility of dietary fibre, saturated fat and energy intake was also assessed with correlation coefficients of 0.61, 0.72 and 0.73 respectively [21]. The dietary questionnaire was completed at registration for the study (t = R), at commencement of the study (on average 13 months later, t = 0) and then at 0.5, 1, 2, 3, 4, 6, 8, and 10 years later.

All completed food questionnaires were linked to Swedish food composition tables to provide nutrient information. For this analysis, 39 food groups were defined based on nutrient content and usual culinary usage (Table S1). Average intakes of each food group (g/day) were calculated at each time-point and standardised relative to data collected at study registration ((Intake – mean (t = R) )/SD(t = R)).

### Dietary Patterns

Dietary patterns were firstly derived using available data collected at registration (n = 6869). The RRR model (PROC PLS in Statistical Analysis Software (version 9.3, SAS Institute Inc)) included dietary energy density (DED), fibre density (FD) and saturated fat intake (% of total energy) as response variables. These variables are the focus of current recommendations from healthcare bodies and governments for weight-loss and improving diet quality conducive to cardiovascular health [23], [24], [25]. The 39 food groups were used as predictors.

Energy density (kJ/g) was calculated as food energy (kJ) divided by food weight (g). Food was defined as solid food and liquids consumed as food (for example, soups and yoghurt). All beverages, both energy-containing (alcoholic drinks, milk, sweetened drinks and fruit juices) and non-energy-containing (water, coffee and diet beverages), were excluded from this calculation [26]. The percentage energy from saturated fat was calculated by dividing daily energy from saturated fat (kJ) by total daily energy intake (kJ) then multiplying by 100. FD (mg/kJ) was calculated by dividing dietary fibre intake (mg) by total energy intake (kJ).

Three dietary patterns were extracted at t = R. Every subject received a z-score for the dietary pattern to quantify how their reported dietary intake reflected the pattern. Dietary patterns were similarly derived at each follow-up to ensure no new patterns appeared during the 10-year follow-up; no new patterns were identified. Only the dietary pattern explaining most variation in response variables was of interest.

To investigate tracking of the same dietary pattern over the 10-year follow up, an applied dietary pattern z-score was calculated for t = 0, 0.5, 1, 2, 3, 4, 6, 8, and 10 years using the scoring coefficients produced by the RRR analysis of registration data [6], [27]. The applied z-score reflected adherence to the dietary pattern observed at registration. This consistency in measurement over time provides a better variable for longitudinal measurement than using z-scores from similar dietary patterns derived from an exploratory analysis conducted at each time point.

### Statistical Analyses

Tracking (or stability) coefficients were calculated for dietary pattern z-scores using a generalised estimating equation (GEE) model [28].

The model regressed repeated measurements of the DP z-score against the DP z-score at registration, adjusting for time between each measurement. As the relationship between the initial DP score and subsequent DP scores is tested simultaneously, the standardised regression coefficient (β*_1_*) can be interpreted as a tracking coefficient [28]. Adjustments were included for time-dependent covariates and time-independent covariates.

GEE was carried out using the GENMOD procedure in SAS v9.3. As dietary intake is included as z-scores, the standardised tracking coefficient ranges from 0 to 1, with 1 indicating perfect tracking and 0 indicating no tracking. There are no universally accepted cut offs to classify good or poor tracking, as the magnitude of the tracking coefficient can depend on the length of follow up and measurement error in the variable being tracked [28]. However, it is possible to contrast tracking coefficients observed within the same study, and we considered weak tracking coefficients as ≤0.3; moderate tracking 0.3–0.6; and high tracking ≥0.6 [29], [30].

**Figure 1:**
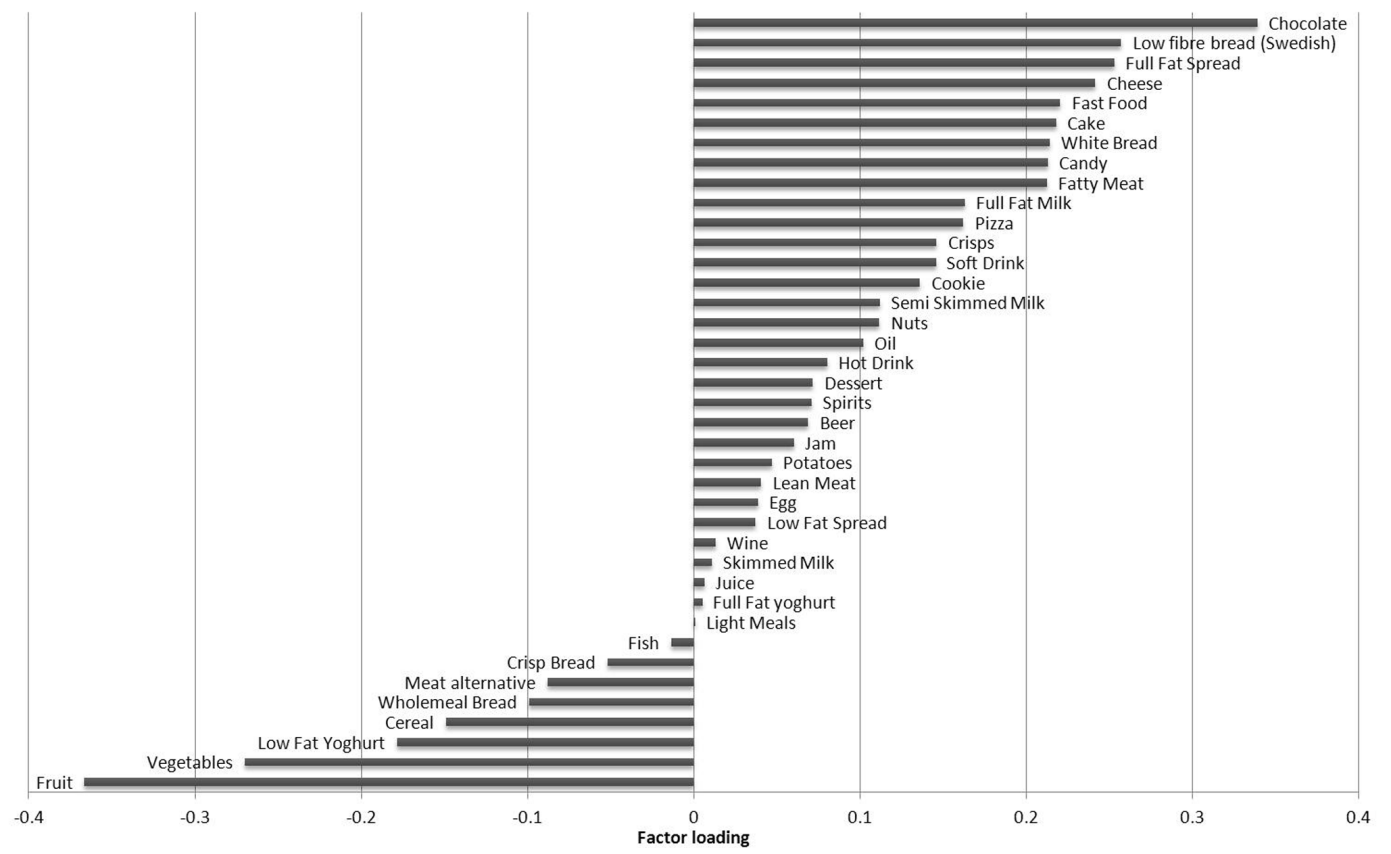
Factor loadings for the first dietary pattern high in energy density, high in percentage saturated fat and low in fibre density.

Interactions between the tracking coefficient and gender were observed (p<0.0001) and so tracking coefficients were estimated separately for men and women. To test the effect of different durations of follow-up, tracking coefficients were estimated between registry (t = R) and 10-years, baseline (t = 0) and 10-years, from 2 to 10-years and from 6 to 10-years. Age, and smoking were examined as time-varying covariates. A similar GEE model was applied to estimate tracking coefficients for key food group intakes and the macronutrients chosen as response variables, which were included in the models as standardised intakes (z-scores). Only those food groups with a factor loading ≥0.2 or ≤ −0.2 were examined (Figure 1).

Because of potential differences in dietary habits and dietary reporting, tracking analyses were conducted separately for men and women. Only those respondents who completed a food diary on at least two follow ups were included in the analyses.

## Results

A total of 2037 participants had dietary data from at least two follow-ups with data from 2037, 1771, 1766, 1667, 1544, 1477, 1364, 1219 and 1131 individuals at t = 0, 0.5, 1, 2, 3, 4, 6, 8, 10 respectively. At registration, the study population had a greater proportion of women (71%) than men (29%) and a mean age of 47.6±6.2 and 46.8±5.8 years respectively for women and men (Table 1). BMI was higher in women (41.6±4.2 kg/m^2^) than men (39.2±4.12 kg/m^2^) due to the SOS inclusion criteria [31].

**Table 1:** Mean (±SD) measures and dietary characteristics of the 2037 individuals at their registration to the study (t = R).

Measure		
	Male	Female
	*n = 590*	*n = 1447*
Age (yrs)	46.8±5.8	47.6±6.2
Smoking (%)	23.7	18.8
Weight (kg)	126.8±14.4	112.9±13.9
Height (m)	1.80±0.07	1.65±0.06
BMI (kg/m^2^)	39.2±4.1	41.6±4.2
Total Energy intake (kJ)	13240±4802	11334±4697
Saturated fat intake (%)	16.0±3.1	16.5±3.1
Fibre density (mg/kJ)	1.93±0.60	2.24±0.62
Dietary energy density (kJ/g)	7.63±1.55	7.12±1.55

The first of the three derived dietary patterns explained most of the variance in the response variables (54%). The remaining two patterns combined explained only 26% of the total variation in response variables and were therefore not included in the tracking analysis. The first pattern explained 31% of the variance in percentage energy from saturated fat, 60% of the variance in fibre density and 71% of the variance in dietary energy density. This pattern was negatively correlated with fibre density (r = −0.61) and positively correlated with percentage energy from saturated fat (r = 0.44) and dietary energy density (r = 0.66). It was therefore labelled an ‘energy-dense, high saturated fat, low fibre dietary pattern’. A high dietary pattern z-score was characterised by higher intakes of chocolate, low-fibre bread, cheese, fast food and cake and lower intakes of fruit and vegetables (Figure 1).

The tracking coefficient (β_1_) for the energy-dense, high saturated fat, low fibre dietary pattern over the entire study period was moderate, at 0.40 (95% CI: 0.38–0.42) for women and 0.38 (95% CI: 0.35–0.41) for men. The DP tracking coefficients were stronger when follow up time was shorter in women (Table 2). Tracking coefficients were consistently stronger in women than men.

**Table 2:** Tracking coefficient and 95% CI of a standardised dietary pattern score, reported food intake and macronutrient intake of 2037 severely obese Swedish men and women.

	Men		Women	
	Tracking coefficient	95% CI	Tracking coefficient	95% CI
***Dietary pattern score (time points)***				
Dietary Pattern score (R-10)	0.38*	0.35–0.41	0.40*	0.38–0.42
Dietary pattern score (0–10)	0.39*	0.36–0.41	0.45*	0.43–0.47
Dietary Pattern score (2–10)	0.45*	0.31–0.49	0.50*	0.44–0.55
Dietary Pattern score (6–10)	0.47*	0.38–0.57	0.53*	0.46–0.61
***Food groups (t = R to 10)***				
Vegetables	0.52	0.48–0.56	0.37	0.35–0.39
Fruit	0.46	0.43–0.49	0.36	0.34–0.38
Chocolate	0.23	0.21–0.25	0.25	0.24–0.27
Swedish sweet bread (low-fibre)	0.25	0.23–0.27	0.28	0.27–0.29
Full Fat spread (butter)	0.19	0.09–0.29	0.25	0.21–0.30
Cheese	0.28	0.25–0.30	0.20	0.18–0.21
Fast Food	0.25	0.22–0.27	0.14	0.13–0.15
Cake	0.21	0.19–0.23	0.24	0.23–0.25
White Bread	0.24	0.19–0.30	0.26	0.22–0.30
Fatty Meat	0.23	0.20–0.25	0.27	0.25–0.28
Candy	0.10	0.08–0.12	0.18	0.17–0.20
***DP response variables (t = R to 10)***				
Total Energy	0.38*	0.36–0.41	0.38*	0.36–0.39
Saturated fat %	0.37	0.35–0.40	0.35	0.33–0.37
Fibre density	0.45	0.41–0.48	0.47	0.45–0.49
Dietary Energy Density	0.35	0.33–0.38	0.38	0.36–0.39

*Adjusted for age and smoking. All other coefficients are adjusted for age, smoking and total energy intake.

Tracking coefficients for the food group intakes were generally lower (Table 2). The highest coefficients for men and women were for vegetable (β_1_ = 0.52 [95% CI: 0.48–0.56] and β_1_ = 0.37 [95% CI: 0.35–0.39] respectively) and fruit (β_1_ = 0.46 [95% CI: 0.43–0.49] and (β_1_ = 0.36 [95% CI: 0.34–0.38]) intake. The lowest coefficients were for intake of fast food in women (β_1_ = 0.14 [95% CI: 0.13–0.15]) and candy in men (β_1_ = 0.10 [95% CI: 0.08–0.12]). Tracking coefficients for macronutrient response variables were similar to the DP ranging from 0.35 to 0.47. The tracking coefficients for macronutrients and foods did not alter with different follow-up times (not shown).

**Figure 2:**
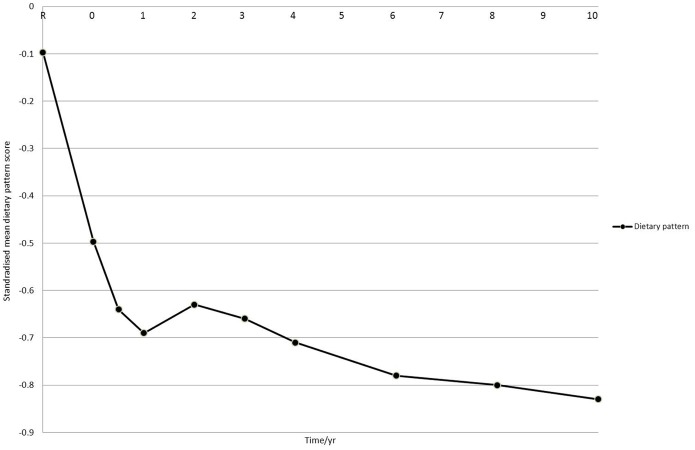
Mean dietary pattern z-score* over 10 years. * z-scores from t0 – t10 were standardised to intakes at t = R.

**Figure 3:**
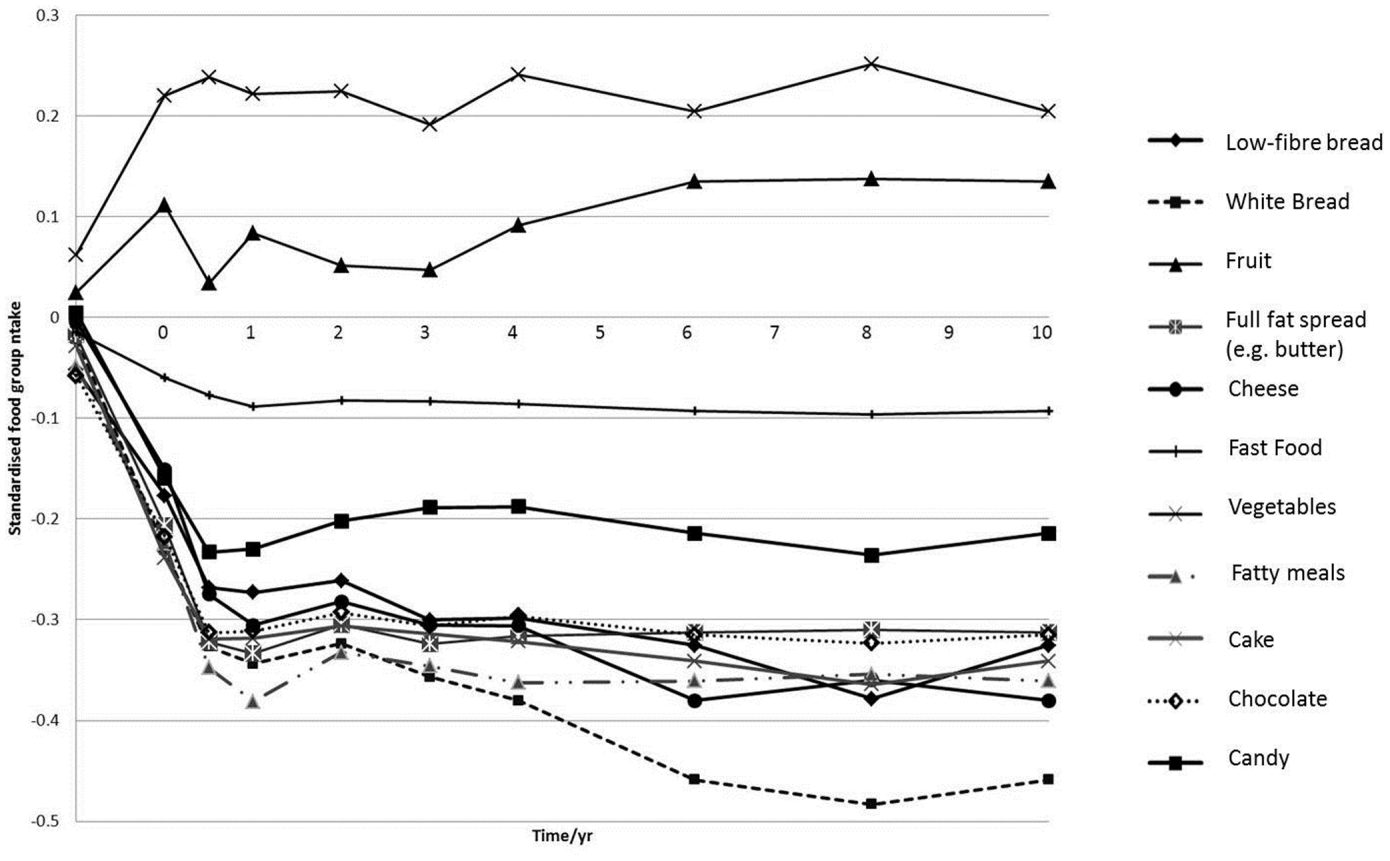
Mean intakes of standardised food group z-scores* over 10 years. *z-scores from t0– t10 were standardised to intakes at t = R.

**Figure 4:**
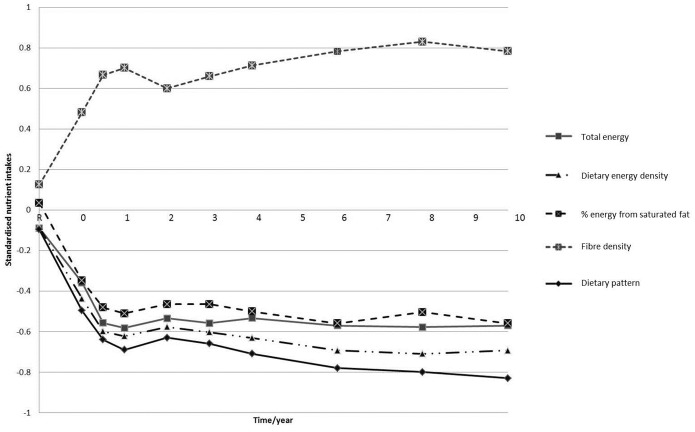
Mean dietary pattern z-score and mean total energy intake, percentage energy from saturated fat, dietary energy density and fibre density z-scores* over 10 years. *z-scores from t0– t10 were standardised to intakes at t = R.

Population mean z-scores for the dietary pattern over 10-years are displayed in Figure 2. Between study registration and one year after baseline, there was a marked decrease in z-score. Population mean z-scores for food groups (Figure 3) and macronutrients (Figure 4) were also plotted. The observed changes in mean intakes correspond with the changes observed in dietary pattern scores.

## Discussion

In this cohort of severely obese adults, an energy dense, high saturated fat, low fibre dietary pattern showed moderate levels of tracking over a 10-year period. However, intakes of food groups important to this dietary pattern showed varying degrees of tracking. This suggests that dietary intake among severely obese adults is not fixed and may be subject to change.

The differences in tracking among food groups indicate that some food intakes are less stable over time, than others. The weaker tracking coefficients for fast food and candy highlight that these foods are more susceptible to change than others and this weaker tracking may be in part due to repeated attempts to lose weight and eliminate these ‘treat’ foods. While food groups with weaker levels of tracking are likely appropriate, modifiable targets for interventions, they may also represent food groups where changes are short term and difficult to maintain. Fruit and vegetable intakes had stronger tracking coefficients, a possible result of strong habit formation and food preference. This is consistent with another study that showed while men made a number of positive dietary changes as part of a weight-loss intervention, increased vegetable intake was least likely [32].

The changes observed in food intakes in this cohort may be explained by a number of factors. For instance, in the waiting period between registration and baseline, participants showed improvements in cardiovascular disease risk factors [33]. This is likely to be a ‘study effect’ and may also be responsible for initial dietary changes as individuals’ have demonstrated a willingness to change by expressing interest in the study. Secondly, it may be that diagnosis of disease may be a pre-cursor to dietary changes in this high risk population. For example, despite receiving no intervention, the control group has demonstrated a rate of recovery from diabetes at 2 years of 21% and at 10 years of 13% [20]. It is likely these recoveries are influenced by dietary and lifestyle changes.

To our knowledge, no other study has looked at the tracking of dietary patterns over time in severely obese adults. Two studies have investigated the tracking of nutrient and food intakes in the Amsterdam Growth and Health Study (AGHS) using similar methods. Post *et al.* [7] analysed repeated dietary measures in 200 individuals over a period of 20 years spanning adolescence and adulthood. The tracking coefficients for total energy (β1 = 0.33; 0.22–0.4) and percentage energy from saturated fat (β1 = 0.41; 0.32–0.50) were similar to that observed in our analysis. In another analysis of AGHS data, Velde *et al*. tracked fruit and vegetable intake in 168 men and women between the ages of 12 and 36 years [34]. Their tracking coefficient for fruit was 0.33 (95% CI: 0.25–0.41); similar to the tracking coefficient for SOS women but weaker than SOS men. Velde’s tracking coefficient for vegetables was 0.27 (95% CI: 0.19–0.36), much lower than that of the current study [34]. However, these studies are not directly comparable as participant numbers, population type and length between follow up measurements may influence tracking coefficients [1]. Despite being over a longer follow-up, the studies in the AGHS, had fewer participants and measurements. This may contribute to the differences observed between the two studies.

This is one of very few studies to track a dietary pattern that was identified using reduced rank regression. The SOS study is one of the largest cohorts of severely obese individuals with repeated dietary measures collected using consistent data collection methods. Despite this, some study limitations should be noted. Firstly, it is possible the categorisation of food groups has influenced the degree to which foods appear to track. For example, it may be easier to swap intake of foods in the ‘Candy’ group with foods in another food group than it is for groups such as fruits or vegetables. We cannot rule out recall bias or dietary misreporting, which is common, to some degree, to all dietary assessments, and this may have influenced some tracking coefficients. However, the dietary assessment in the SOS study was adapted for the severely obese population to minimise biases due to the nature of the population. The levels of under-reporting in the SOS dietary assessment tool are no greater than those seen in the general population [21]. As the study occurred over ten years, a potential limitation is loss to follow up as is frequently observed in longitudinal studies. The loss to follow-up was greatest at 10-years with a 56% loss. However, this remains a large cohort of severely obese individuals at 10-years with 1130 responses. Furthermore, the application of longitudinal models to estimate the tracking coefficients utilised all available data at each follow up, thus avoiding some of the potential bias associated with ‘completers only’ analyses. By comparison, most studies reporting dietary tracking have been limited to only two measurements over time and the use of more simple statistical methods e.g. Pearson’s correlation and measures of agreement (e.g. Kappa statistics) as indicators of dietary tracking [35]. Finally, while we considered the influence of gender, age and smoking, it is possible that there are other important influences on dietary tracking that were not considered in this study.

The varying degrees of tracking of diet in this population, highlights the importance of repeated measures in cohort studies. A single measure of diet at baseline is likely to be a poor proxy for total diet over later years or decades. Further work is needed in a variety of cohorts to better understand the stability of diet in different adult populations.

In conclusion, an energy dense, high saturated fat, low fibre density dietary pattern and selected food and nutrient intakes track moderately over a 10-year period in this cohort of severely obese adults. The intakes of some food groups appear more subject to change than others while some, often the most healthful, appear more stable and may require earlier intervention. While there is continued scepticism about the long-term effectiveness of lifestyle interventions in the severely obese [36], [37], [38], [39], these results suggest that while changes in some foods are likely to be more successful than others, there are opportunities to change dietary habits in the severely obese, which deserve further investigation.

## Supporting Information

Table S1 **SOS Food groups and their constituents.**
(DOC)

## References

[1] Twisk JWR (2009) Applied longitudinal data analysis for epidemiology: a practical guide. Cambridge: Cambridge University Press.

[2] Totland TH, Gebremariam MK, Lien N, Bjelland M, Grydeland M, et al (2013) Does tracking of dietary behaviours differ by parental education in children during the transition into adolescence? Public Health Nutrition 16: 673–682. 22874120 10.1017/S1368980012003060 PMC10271322

[3] Demory-Luce D, Morales M, Nicklas T, Baranowski T, Zakeri I, et al (2004) Changes in food group consumption patterns from childhood to young adulthood: The Bogalusa Heart Study. Journal of the American Dietetic Association 104: 1684–1691. 15499355 10.1016/j.jada.2004.07.026

[4] Wang YF, Bentley ME, Zhai FY, Popkin BM (2002) Tracking of dietary intake patterns of Chinese from childhood to adolescence over a six-year follow-up period. J Nutr 132: 430–438. 11880567 10.1093/jn/132.3.430

[5] Li J, Wang YF (2008) Tracking of dietary intake patterns is associated with baseline characteristics of urban low-income African-American adolescents. Journal of Nutrition 138: 94–100. 18156410 10.1093/jn/138.1.94

[6] Ambrosini GL, Emmett PM, Northstone K, Jebb SA (2013) Tracking a dietary pattern associated with increased adiposity in childhood and adolescence. Obesity: n/a-n/a. 10.1002/oby.20542 PMC3846445 23804590

[7] Post GB, de Vente W, Kemper HCG, Twisk JWR (2001) Longitudinal trends in and tracking of energy and nutrient intake over 20 years in a Dutch cohort of men and women between 13 and 33 years of age: The Amsterdam growth and health longitudinal study. Br J Nutr 85: 375–385. 11299083 10.1079/bjn2000249

[8] Lien N, Lytle LA, Klepp K-I (2001) Stability in Consumption of Fruit, Vegetables, and Sugary Foods in a Cohort from Age 14 to Age 21. Preventive Medicine 33: 217–226. 11522162 10.1006/pmed.2001.0874

[9] Borland SE, Robinson SM, Crozier SR, Inskip HM (2007) Stability of dietary patterns in young women over a 2-year period. European Journal of Clinical Nutrition 62: 119–126. 17299458 10.1038/sj.ejcn.1602684

[10] Mikkilae V, Rasnan L, Raitakari OT, Marniemi J, Pietinen P, et al (2007) Major dietary patterns and cardiovascular risk factors from childhood to adulthood. The Cardiovascular Risk in Young Finns Study. British Journal of Nutrition 98: 218–225. 17367571 10.1017/S0007114507691831

[11] Prevost AT, Whichelow MJ, Cox BD (1997) Longitudinal dietary changes between 1984–5 and 1991–2 in British adults: associations with socio-demographic, lifestyle and health factors. British Journal of Nutrition 78: 873–888. 9497440 10.1079/bjn19970206

[12] Mokdad AH, Bowman BA, Ford ES, Vinicor F, Marks JS, et al (2001) The continuing epidemics of obesity and diabetes in the United States. Jama-Journal of the American Medical Association 286: 1195–1200. 10.1001/jama.286.10.1195 11559264

[13] Hjartaker A, Laake P, Lund E (2001) Body mass index and weight change attempts among adult women - The Norwegian Women and Cancer Study. European Journal of Public Health 11: 141–146. 11420799 10.1093/eurpub/11.2.141

[14] Ashima K K (2004) Dietary patterns and health outcomes. Journal of the American Dietetic Association 104: 615–635. 15054348 10.1016/j.jada.2004.01.010

[15] Crozier SR, Robinson SM, Godfrey KM, Cooper C, Inskip HM (2009) Women’s Dietary Patterns Change Little from Before to During Pregnancy. Journal of Nutrition 139: 1956–1963. 19710161 10.3945/jn.109.109579 PMC3113465

[16] Mishra GD, McNaughton SA, Bramwell GD, Wadsworth MEJ (2006) Longitudinal changes in dietary patterns during adult life. British Journal of Nutrition 96: 735–744. 17010234

[17] Weismayer C, Anderson JG, Wolk A (2006) Changes in the stability of dietary patterns in a study of middle-aged Swedish women. Journal of Nutrition 136: 1582–1587. 16702325 10.1093/jn/136.6.1582

[18] Hoffmann K, Schulze MB, Schienkiewitz A, Nothlings U, Boeing H (2004) Application of a new statistical method to derive dietary patterns in nutritional epidemiology. Am J Epidemiol 159: 935–944. 15128605 10.1093/aje/kwh134

[19] Hoffmann K, Schulze MB, Schienkiewitz A, Nothlings U, Boeing H (2004) Application of a new statistical method to derive dietary patterns in nutritional epidemiology. American Journal of Epidemiology 159: 935–944. 15128605 10.1093/aje/kwh134

[20] Sjostrom L, Lindroos AK, Peltonen M, Torgerson J, Bouchard C, et al (2004) Lifestyle, diabetes, and cardiovascular risk factors 10 years after bariatric surgery. New England Journal of Medicine 351: 2683–2693. 15616203 10.1056/NEJMoa035622

[21] Lindroos AK, Lissner L, Sjostrom L (1993) Validity and Reproducibility of a Self-Administered Dietary Questionnaire in Obese and Nonobese Subjects. European Journal of Clinical Nutrition 47: 461–481. 8404782

[22] Lindroos AK, Lissner L, Sjostrom L (1999) Does degree of obesity influence the validity of reported energy and protein intake? Results from the SOS Dietary Questionnaire. European Journal of Clinical Nutrition 53: 375–378. 10369492 10.1038/sj.ejcn.1600732

[23] U.S. Department of Agriculture and U.S. Department of Health and Human Services (2010) Dietary Guidelines for Americans, 2010. Washington, DC: U.S. Government Printing Office.

[24] NHS Choices Available: http://www.nhs.uk/livewell/goodfood/Pages/Goodfoodhome.aspx Accessed 2014 Apr 25.

[25] American Dietetic Association Available: http://www.eatright.org/public/default.aspx Accessed 2014 Apr 25.

[26] Johnson L, Mander AP, Jones LR, Emmett PM, Jebb SA (2008) Energy-dense, low-fiber, high-fat dietary pattern is associated with increased fatness in childhood. American Journal of Clinical Nutrition 87: 846–854. 18400706 10.1093/ajcn/87.4.846

[27] Imamura F, Lichtenstein AH, Dallal GE, Meigs JB, Jacques PF (2009) Generalizability of dietary patterns associated with incidence of type 2 diabetes mellitus. The American Journal of Clinical Nutrition 90: 1075–1083. 19710193 10.3945/ajcn.2009.28009 PMC2744626

[28] Twisk JWR, Kemper HCG, Mellenbergh GJ, van Mechelen W, Post GB (1996) Relation between the longitudinal development of lipoprotein levels and lifestyle parameters during adolescence and young adulthood. Annals of Epidemiology 6: 246–256. 8827160 10.1016/1047-2797(96)00003-8

[29] Pearson N, Salmon J, Campbell K, Crawford D, Timperio A (2011) Tracking of children’s body-mass index, television viewing and dietary intake over five-years. Preventive Medicine 53: 268–270. 21820008 10.1016/j.ypmed.2011.07.014

[30] Twisk JWR, Kemper HCG, van Mechelen W, Post GB (1997) Tracking of Risk Factors for Coronary Heart Disease over a 14-Year Period: A Comparison between Lifestyle and Biologic Risk Factors with Data from the Amsterdam Growth and Health Study. American Journal of Epidemiology 145: 888–898. 9149660 10.1093/oxfordjournals.aje.a009048

[31] Sjostrom L, Backman L, Bengtsson C, Dahlgren S, Jonsson E, et al (1987) Announcement of the Multicenter Project Swedish Obese Subjects-Sos. International Journal of Obesity 11: 87–87.3032830

[32] Collins CE, Morgan PJ, Warren JM, Lubans DR, Callister R (2010) Men participating in a weight-loss intervention are able to implement key dietary messages, but not those relating to vegetables or alcohol: the Self-Help, Exercise and Diet using Internet Technology (SHED-IT) study. Public Health Nutrition 14: 168–175. 20602869 10.1017/S1368980010001916

[33] Sjostrom L, Lindroos AK, Peltonen M, Torgerson J, Bouchard C, et al (2004) Lifestyle, diabetes, and cardiovascular risk factors 10 years after bariatric surgery. N Engl J Med 351: 2683–2693. 15616203 10.1056/NEJMoa035622

[34] Velde SJ, Twisk JWR, Brug J (2007) Tracking of fruit and vegetable consumption from adolescence into adulthood and its longitudinal association with overweight. British Journal of Nutrition 98: 431–438. 17433126 10.1017/S0007114507721451

[35] Northstone K, Emmett PM (2008) A comparison of methods to assess changes in dietary patterns from pregnancy to 4 years post-partum obtained using principal components analysis. British Journal of Nutrition 99: 1099–1106. 17916275 10.1017/S0007114507842802 PMC2493053

[36] Gondoni LA, Liuzzi A (2011) Diet and Physical Activity Interventions in Severely Obese Adults. Journal of the American Medical Association 305: 563. 10.1001/jama.2011.82 21304077

[37] Goodpaster BH, DeLany JP, Jakcic JM (2011) Diet and Physical Activity Interventions in Severely Obese Adults Reply. Journal of the American Medical Association 305: 564–564.21304078

[38] Goodpaster BH, DeLany JP, Otto AD, Kuller L, Vockley J, et al (2010) Effects of Diet and Physical Activity Interventions on Weight Loss and Cardiometabolic Risk Factors in Severely Obese Adults A Randomized Trial. Journal of the American Medical Association 304: 1795–1802. 20935337 10.1001/jama.2010.1505 PMC3082279

[39] Hemmingsson E, Udden J, Rossner S (2011) Diet and Physical Activity Interventions in Severely Obese Adults. Journal of the American Medical Association 305: 563–564. 10.1001/jama.2011.83 21304076

