# Supplementary material for: Tracking of a Dietary Pattern and Its Components over 10-Years in the Severely Obese
Source: PLoS One. 2014 May 19;9(5):e97457. doi: 10.1371/journal.pone.0097457 (PMC4026226; doi:10.1371/journal.pone.0097457)
Supplement: Table S1 — SOS Food groups and their constituents. (DOC) [file pone.0097457.s001.doc]

Food group Questionnaire level foods and food groups included

Fruit Citrus fruit, apples/pears, bananas, grapes, plums, melon, peaches/nectarines, exotic fruits (kiwi, mango, pineapple).

Vegetables Vegetables

Cereal Porridge, gruel, breakfast cereal

Wholemeal Bread Rye, whole-wheat, brown bread

White Bread White bread

Crisp bread Crisp bread

Low fibre bread (Swedish) Swedish sweet bread

Low Fat Spread Low-fat margarine

Full Fat Spread Butter, mixture of butter and oil , margarine

Skimmed Milk Skimmed milk and low fat milk (0.5% fat)

Semi Skimmed Milk Low fat milk (1% fat)

Full Fat Milk Whole milk (3% fat)

Full Fat yoghurt Yoghurt/fermented milk (Whole – 3% fat)

Low Fat Yoghurt Low fat yoghurt (0.5% fat)

Juice Pure fruit juice

Soft Drink Fruit drink (squash), soft drink

Wine Wine, Sherry, dessert wines

Beer Beer

Spirits Liquor (Spirits, cognac, whisky etc)

**Supplemental Table 1: Continued**

Food group Questionnaire level foods and food groups included

Hot Drinks Coffee, tea, hot (or cold) chocolate drink (incl milk)

Potatoes Potatoes, rice, spaghetti etc

Meat alternative Soy burgers, lentils, beans etc.

Fish Fish, seafood

Lean Meat beef, ground meat, pork, chicken, fowel

Fatty Meat Salted pork, bacon, sausage, other fatty meats

Oils Cooking oil, butter, margarine

Light Meals Light meals (Omelettes, soup, salad, hot sandwiches)

Fast Food Fast food (Hamburgers, hot dogs, fries)

Pizza Pizza

Dessert Fruit dessert, ice-cream, pie, pudding, chocolate mousse

Egg Egg

Jam Jam spread

Cheese Cheese, cottage cheese, cheese spread

Nuts Nuts, Pretzels

Chocolate Chocolate bars and box chocolates

Cookies Cookies and biscuits

Crisps Crisps, Cheese puffs etc.

Candy Non-chocolate sweets, Candies or equivalent

Cake High fat, high sugar cakes, Sweet buns, Swedish sweet cakes
